# Evaluation of Surgical Cases of Lung Cancer Admitted in Shiraz Referral Hospitals, Southern Iran in 2009–2022

**DOI:** 10.1002/cnr2.70108

**Published:** 2025-03-12

**Authors:** Sara Dehghani, Alireza Rezvani, Reza Shahriarirad, Mohammad Sadegh Rajabian, Bizhan Ziaian, Mohammad Javad Fallahi, Parviz Mardani, Armin Amirian

**Affiliations:** ^1^ Thoracic and Vascular Surgery Research Center Shiraz University of Medical Science Shiraz Iran; ^2^ Student Research Committee Shiraz University of Medical Sciences Shiraz Iran; ^3^ Bone Marrow Transplantation Center, Nemazi Hospital Shiraz University of Medical Sciences Shiraz Iran; ^4^ Department of Surgery Shiraz University of Medical Sciences Shiraz Iran; ^5^ Department of Internal Medicine Shiraz University of Medical Sciences Shiraz Iran

**Keywords:** carcinoma, epidemiology, lung neoplasms, metastasectomy, non‐small‐cell lung, thoracic surgery

## Abstract

**Introduction:**

Globally, lung cancer is one of the most commonly diagnosed cancers and continues to take the lead in cancer‐related mortality rates. This study aims to provide the latest statistics on the clinical, histopathological, and epidemiological features of lung cancer patients who underwent surgical resection in referral hospitals in Southern Iran.

**Method:**

In this retrospective study, records of all patients with operable primary and secondary lung cancer who underwent surgical resection of the lung in Shiraz hospitals, located in Southern Iran from November 2009 to May 2022 were screened. Data on demographic, clinical, surgical, and pathological characteristics were analyzed by SPSS software.

**Results:**

A total of 232 patients with operable lung cancer, including 150 (64.7%) primary cases and 82 (35.3%) secondary cases, underwent 249 operations. The mean age of primary and secondary lung cancer patients was 56.70 ± 13.99 and 45.56 ± 18.88, respectively (*p* < 0.001). Males accounted for 54.0% and 58.5% of primary and secondary lung cancer patients, respectively. Adenocarcinoma was the most frequent primary pathology, while sarcomas were the most common metastatic lesions. The predominant presenting symptoms were cough (*n* = 75, 75.0%) and dyspnea (*n* = 31, 59.7%) in primary and secondary cases, respectively. Involvement of the right lung was more frequent in both groups (65.5% and 53.1% for primary and secondary cases respectively). The most commonly performed surgeries were lobectomy (69.9%) and limited resection (69.8%) for primary and secondary lesions, respectively. Cigarette smoking and extensive resection had a significant association with the in‐hospital mortality rate (*p* = 0.012 and 0.009 respectively). The overall in‐hospital mortality rate was 3.6% (*n* = 9).

**Conclusion:**

Surgical interventions were mostly performed in men and histopathologic subtypes of primary lung adenocarcinoma, metastatic soft tissue sarcoma, and metastatic colon cancer. Smoking and extensive resection accompany a higher risk of short‐term postoperative mortality.

## 1 Introduction

Lung cancer is one of the most frequently diagnosed cancers and continues to take the lead in cancer‐related mortality rates around the world [1]. According to GLOBOCAN 2020, lung cancer ranked third in terms of cancer incidence and second in terms of cancer mortality rates in Iran, accounting for 8% of all new cancer cases and 11.5% of all cancer‐related deaths [2]. Moreover, its incidence and mortality rates showed an increase in recent decades in Iran, and it is projected to experience a significant rise in the future, similar to other low‐ and middle‐income countries. Thus, the instant implementation of policies is fundamental in managing and controlling the disease [3].

Surgical resection is considered the only potentially curative treatment modality for various types of lung malignancies [4, 5]. It remains the standard care for early‐stage non‐small cell lung cancer (NSCLC) and is also part of multidisciplinary management approach for advanced stages of NSCLC and other pathologic subtypes in certain cases [4]. Lungs are also one of the most common targets of metastasis from other organs [6]. While patients with metastatic malignancies carry a poor prognosis without treatment, pulmonary metastasectomy offers a valuable therapeutic option in selected cases, improving patient survival [5]. Recent developments in minimally invasive surgery like video‐assisted thoracic surgery (VATS) have improved surgical outcomes [7]. Furthermore, segmentectomy has emerged as an acceptable alternative to lobectomy for certain early‐stage NSCLC cases, preserving more lung function while achieving comparable oncological outcomes [8].

Besides surgery, neoadjuvant therapies help downstage tumors and increase their resectability. Neoadjuvant chemotherapy is a common practice in patients with locally advanced carcinoma before surgery. The introduction of targeted therapies and immune check point inhibitors has revolutionized the landscape of lung cancer treatment, improving survival rates [9].

Epidemiological studies have shown a global geographical disparity in demographical and clinical characteristics of lung cancer patients; these differences were also noted at a subnational level within Iran [3, 10]. Additionally, a transformation in trends of lung cancer epidemiology, histopathologic features, and treatment modalities is happening worldwide, which affects the characteristics of surgical cases as well [11]. Thus, we aimed to appraise the epidemiological, clinical, and surgical features of patients who received surgical care for primary and metastatic lung cancer in the referral hospitals of Shiraz, located in Southern Iran, between 2009 and 2022.

## 2 Materials and Methods

In this retrospective study, we evaluated the hospital records of patients with operable primary or secondary lung cancer who underwent surgical resection of the lung for therapeutic intent in Shiraz referral hospitals, located in Southern Iran, from November 2009 to May 2022. The data were collected from three major hospitals with thoracic surgery departments in Shiraz including Namazi, Kowsar, and Abu‐Ali Sina hospitals. Patients who underwent surgery for diagnostic purposes or had inoperable tumors after thoracotomy were excluded from the study.

The records were carefully reviewed by referring to the hospital archives and specifying the records according to the specific disease code. Information related to lung cancer, including demographic data such as age, sex, place of residence, as well as the type of lung cancer, clinical symptoms, epidemiological factors, surgical interventions, and all other information related to the disease, were extracted from these patients' records. The extracted information was then entered in the special data sheet and analyzed using SPSS version 26.0 software. Descriptive data are presented as frequency and percentage, while numerical data are presented as either mean and standard deviation (SD), or median and interquartile range [IQR], based on their parametric distribution. The relationship between lung cancer and the associated factors was evaluated using either the Chi‐square test (*χ* ^2^) for categorical variables or the independent sample *t*‐test/Mann–Whitney U test for continuous variables. A *p*‐value of less than 0.05 was considered as the significance level.

## 3 Results

**FIGURE 1:**
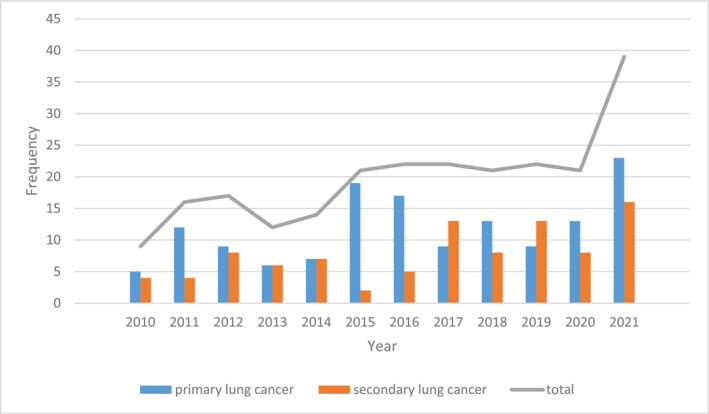
The annual rate of lung cancer surgeries in Shiraz hospitals, Southern Iran.

During the 12.5‐year period of our study, a total of 232 patients with operable primary or metastatic lung malignancies underwent 249 pulmonary resection operations in three main hospitals in Shiraz, Southern Iran. This corresponds to an average annual rate of 18.04 ± 7.07 cases and 19.40 ± 7.17 operations. A higher proportion of operations were performed at Namazi Hospital (55%), followed by Kowsar Hospital (24.9%) and Abu‐Ali Sina Hospital (20.1%). As Figure 1 demonstrates, the annual rate of total lung cancer surgeries increased in 2015 and remained stable until 2020. However, it nearly doubled in 2021 compared to the preceding years.

**FIGURE 2:**
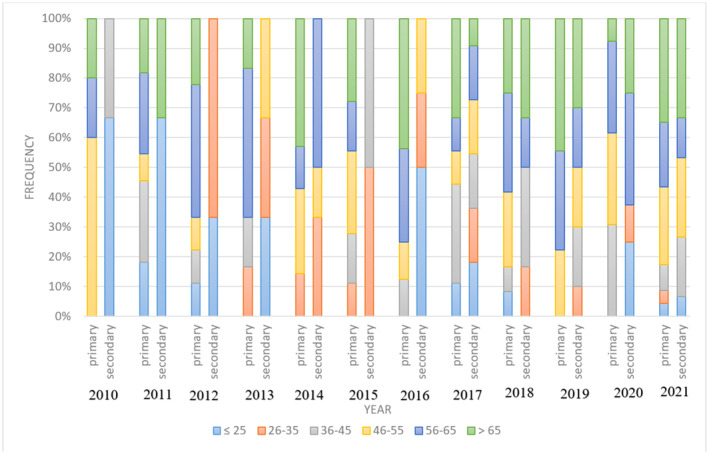
Time trend of age groups in surgical cases of primary and secondary lung cancer in Shiraz hospitals, Southern Iran.

Demographic characteristics of the 232 cases of lung cancer are presented in Table 1. The overall male‐to‐female ratio was 5:4, and the average age of the patients was 52.74 ± 16.74 years (range 18–84). A noticeable increasing trend in prevalence was seen among age groups (*p* < 0.001) and patients with primary lung cancer were mainly older than those with secondary lung cancer (*p* < 0.001). Also, the proportion of patients aged over 65 increased in the second half of the study period for both the primary and metastatic groups (Figure 2).

**TABLE 1:** Demographic characteristics of patients with primary and secondary lung cancer in Southern Iran.

Variable		Total; *N* = 232	Histopathology		*p*‐value
			Primary; *n =* 150	Secondary; *n =* 82	
Age (years); mean ± standard deviation		52.74 ± 16.73	56.70 ± 13.99	45.56 ± 18.88	**< 0.001**
Age group (years); *n* (%)	≤ 25	21 (9.1)	6 (4.0)	15 (18.3)	**< 0.001**
	26–35	21 (9.1)	6 (4.0)	15 (18.3)	
	36–45	31 (13.4)	20 (13.4)	11 (13.4)	
	46–55	43 (18.6)	31 (20.8)	12 (14.6)	
	56–65	52 (22.5)	39 (26.2)	13 (15.9)	
	> 65	63 (27.3)	47 (31.5)	16 (19.5)	
Gender; *n* (%)	Male	129 (55.6)	81 (54.0)	48 (58.5)	0.506
	Female	103 (44.4)	69 (46.0)	34 (41.5)	
Year; *n* (%)	2010–2013	49 (22.4)	31 (22.3)	18 (22.5)	0.440
	2014–2017	74 (33.8)	51 (36.7)	23 (28.8)	
	2018–2021	96 (43.8)	57 (41.0)	39 (48.8)	
Residence; *n* (%)	Capital of province	96 (41.7)	66 (44.3)	30 (37.0)	0.286
	Other	134 (58.3)	83 (55.7)	51 (63.0)	
Social history; *n* (%)	Cigarette smoker	59 (28.6)	52 (39.7)	7 (9.3)	**< 0.001**
	Waterpipe smoker	17 (8.2)	15 (11.3)	2 (2.7)	**0.029**
	Opium consumption	32 (15.5)	28 (21.2)	4 (5.3)	**0.002**
	Alcohol consumption	4 (1.9)	2 (1.5)	2 (2.7)	0.622
Comorbid disease; *n* (%)	Chronic obstructive pulmonary disease	15 (6.5)	15 (10.0)	0 (0)	**0.003**
	Asthma	14 (6.1)	13 (8.7)	1 (1.2)	**0.022**
	Small airway disease	1 (0.4)	1 (0.7)	0 (0)	1.000
	Tuberculosis	1 (0.4)	1 (0.7)	0 (0)	1.000
	Pneumonia	1 (0.4)	1 (0.7)	0 (0)	1.000

*Note:* The *p*‐values were calculated using Chi‐square and Mann–Whitney U test. Bold values indicate significant association.

Cigarette smoking, waterpipe smoking, and opium consumption showed a significant association with primary lesions (*p* < 0.001, *p* = 0.029, and *p* = 0.002, respectively). While the majority of patients were nonsmokers (71.4%), the majority of smokers were males (*n* = 51, 86.4%). Also, 88.1% of smokers had primary lung cancer, including 42.4% with squamous cell carcinoma (SCC), 30.5% with adenocarcinoma, 6.8% with low/intermediate grade neuroendocrine tumor (NET), 5.1% with small cell lung cancer (SCLC) or high‐grade NET, and 3.4% with large cell carcinoma.

Among the patients, 32 (13.79%) reported a past medical history, and those with primary tumors had a significantly higher rate of chronic obstructive pulmonary disease and a history of asthma (*p* = 0.003 and *p* = 0.039, respectively). Additionally, two (9.1%) patients with primary lung cancer had a positive family history of lung cancer in first‐ or second‐degree relatives, although no significant association was detected (*p* = 0.556).

Table 2 represents the histopathologic classification of resected tumors, with 150 (64.7%) lesions originating primarily from lung tissue and 82 (35.3%) identified as metastatic tumors, classified as primary and secondary lung cancer. The most frequent primary pathologies were adenocarcinoma, SCC, and low‐grade and intermediate‐grade NET, respectively. Sarcomas were the most metastatic lesions, followed by colon cancer. Among the primary lesions, only SCC showed a significant association with cigarette smoking (*p* < 0.001).

**TABLE 2:** Histopathological subtypes of primary and secondary lung cancer in surgical cases in Southern Iran.

Type of lung cancer			Frequency (%)
Primary	Total		150 (64.7)
	NSCLC	Total	114 (76.0)
		Adenocarcinoma	73 (48.7)
		Squamous cell carcinoma	39 (26.0)
		Adenosquamous	2 (1.3)
	Neuroendocrine	Total	34 (22.7)
		Small cell lung cancer/high‐grade NET	3 (2.0)
		Large cell lung cancer	6 (4.0)
		NET (low grade and intermediate grade)	25 (16.7)
	Sarcoma		2 (1.3)
Secondary	Total		82 (35.3)
	Metastatic soft tissue sarcoma	Total	18 (22.0)
		Fibrosarcoma	4 (4.9)
		Peripheral nerve sheath tumor	3 (3.7)
		Leiomyosarcoma	2 (2.4)
		Hemangioepithelioma	1 (1.2)
		Myxofibrosarcoma	1 (1.2)
		Myofibroblastic tumor	1 (1.2)
		Liposarcoma	1 (1.2)
	Colon cancer		17 (20.7)
	Osteosarcoma		9 (11.0)
	Germ cell tumor		8 (9.8)
	PNET/Ewing family		5 (6.1)
	Genitourinary tract tumor	Total	4 (4.9)
		Renal cell carcinoma	3 (3.7)
		Papillary urothelial carcinoma	1 (1.2)
	Breast		4 (4.9)
	Rectal cancer		3 (3.7)
	Thyroid cancer	Total	3 (3.7)
		Papillary thyroid carcinoma	1 (1.2)
		Follicular thyroid cancer	1 (1.2)
		Insular carcinoma of the thyroid	1 (1.2)
	Melanoma		2 (2.4)
	Hepatocellular carcinoma		2 (2.4)
	Ovarian cancer		1 (1.2)
	Wilms tumor		1 (1.2)
	Laryngeal SCC		1 (1.2)
	Meningioma of brain		1 (1.2)

	Thymoma		1 (1.2)
	Leiomyoma		1 (1.2)
Mucoepidermal carcinoma of the salivary gland		1 (1.2)

Abbreviations: NET, neuroendocrine tumor; NSCLC, non‐small cell lung cancer; PNET, primitive neuroectodermal tumor; SCC, squamous cell carcinoma.

An overview of clinical and surgical features of patients, as well as the pathological characteristics of the resected lesions from all 256 surgeries is shown in Table 3. In patients with primary lung cancer, the most frequent symptoms at presentation were cough (75.0%) and dyspnea (59.7%), which were significantly higher compared to secondary tumors (*p* < 0.001 and *p* = 0.041, respectively). Furthermore, cough was the presenting symptom in 89.7% of the primary cases with right upper lobe involvement (*p* = 0.031). Hemoptysis was more frequently observed in patients with low and intermediate NET subtype (*p* = 0.002). Additionally, although not significant, weight loss was more frequently reported in patients with metastatic lesions (*p* = 0.206).

**TABLE 3:** Clinical and surgical characteristics of patients with primary and secondary lung cancer in Southern Iran.

Variable			Total; *N = 249*	Type of lung cancer		
				Primary; *n* = 153	Secondary; *n =* 96	*p*‐value
Symptoms; *n* (%)	Dyspnea		102 (54.0)	71 (59.7)	31 (44.3)	**0.041**
	Cough		95 (56.2)	75 (75.0)	20 (29.0)	**< 0.001**
	Hemoptysis		42 (25.5)	31 (31.3)	11 (16.7)	**0.034**
	Chest pain		32 (18.0)	22 (19.8)	10 (14.9)	0.410
	Weight loss		30 (18.3)	9 (13.6)	21 (21.4)	0.206
Location; *n* (%)	Right	Total	143 (57.7)	92 (60.5)	51 (53.1)	0.251
		Upper lobe	78 (31.5)	45 (29.6)	33 (34.4)	0.431
		Middle lobe	49 (19.8)	34 (22.4)	15 (15.6)	0.194
		Lower lobe	83 (33.5)	47 (30.9)	36 (37.5)	0.285
	Left	Total	108 (43.5)	61 (40.1)	47 (49.0)	0.172
		Upper lobe	67 (27.0)	33 (21.7)	34 (35.4)	**0.018**
		Lower lobe	65 (26.2)	37 (24.3)	28 (29.2)	0.400
Multilobar involvement			70 (28.2)	34 (22.4)	36 (37.5)	**0.010**
Largest diameter (cm); median [IQR] or *n* (%)	Total		3.5 [3.2]	4.0 [3.2]	3.0 [3.4]	0.113
	≤ 2		29 (21.5)	11 (14.5)	18 (30.5)	0.076
	2–5		69 (51.1)	43 (56.6)	26 (44.1)	
	> 5		37 (27.4)	22 (28.9)	15 (25.4)	
Area of the lesions (cm^2^); median [IQR]			9 [16]	9.71 [15.56]	8.72 [18.50]	0.737
Pathological features; *n* (%)	Resection marginal involvement		28 (21.4)	14 (16.9)	14 (29.2)	0.098
	Pleural margin involvement		20 (17.4)	16 (17.8)	4 (16.0)	1.000
	Lymphovascular invasion		38 (32.2)	36 (34.6)	2 (14.3)	0.221
	Perineural invasion		9 (8.4)	9 (9.8)	0 (0.0)	0.354

	Stage	I	31 (52.5)	31 (52.5)	—	—
		II	15 (25.5)	15 (25.5)	—	—
		III	9 (15.3)	9 (15.3)	—	—
IV		4 (6.8)	4 (6.8)	—	—
Operative procedure; *n* (%)	Extensive resection		43 (17.3)	36 (23.5)	7 (7.3)	**0.001**
	Pneumonectomy		19 (7.6)	17 (11.1)	2 (2.1)	**0.009**
	Bilobectomy		11 (4.4)	9 (5.9)	2 (2.1)	0.212
	Lobectomy and limited resection		13 (5.2)	10 (6.5)	3 (3.1)	0.239
	Lobectomy		129 (51.8)	107 (69.9)	22 (22.9)	**< 0.001**
	Limited resection		77 (30.9)	10 (6.5)	67 (69.8)	**< 0.001**
	Segmentectomy		13 (21.0)	0 (0.0)	13 (23.6)	0.328
	Wedge resection		43 (69.4)	6 (85.7)	37 (67.3)	0.422
	Segmentectomy and wedge resection		6 (9.7)	1 (14.3)	5 (9.1)	0.528
Hospitalization duration (days); median [IQR]			8 [4.0]	8 [5.0]	8 [3.5]	**0.009**
In‐hospital mortality			9 (3.6)	7 (4.6)	2 (2.1)	0.489

*Note:* The *p*‐values were assessed using Chi‐square and Mann–Whitney U test. Bold values indicate significant association.

Abbreviation: IQR, interquartile range.

Overall, right lung involvement was more prevalent (57.7%), and the left upper lobe was significantly more involved with secondary lesions (OR = 1.978; CI 95%: 1.120–3.493; *p* = 0.018). Further analysis revealed that among patients with secondary lung cancer, males had a significantly higher rate of right lung involvement, while females had a significantly higher rate of left lung involvement (*p* = 0.046 and 0.042, respectively). Multilobar involvement was significantly higher in secondary lung lesions (OR = 2.082; CI95%: 1.187–3.654; *p* = 0.010). Also, the surgery rate was higher in lower pathological stages.

**FIGURE 3:**
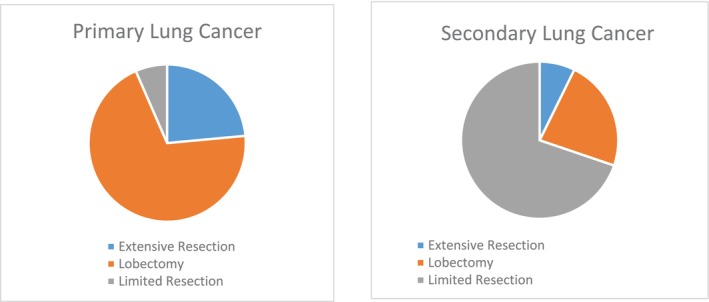
An overview of operative procedures for primary and secondary lung cancer in Shiraz hospitals, Southern Iran.

An overview of operative procedures is illustrated in Figure 3. Among the performed surgeries, 43 (17.3%) were extensive resection, which consisted of pneumonectomy (*n* = 19; 7.6%), bilobectomy (*n* = 11; 4.4%), and lobectomy along with limited resection (*n* = 13; 5.2%). The patients who underwent limited resection (*n* = 77; 30.9%), the most common procedures were wedge resection (*n* = 43; 69.4%), segmentectomy (*n* = 13; 21.0%), and segmentectomy along with wedge resection (*n* = 6; 9.7%). Overall, lobectomy was the most frequently performed resection technique for primary tumors (69.9%), while limited resection was the technique of choice for secondary lung lesions (69.8%).

The size and area of the tumor was significantly larger in the lobectomy group (median [IQR]: size: 3.5 [2.3] cm, area: 10.5 [11.7] cm^2^) compared to the sublobar group (median [IQR]: size: 2.2 [3.4] cm, area: 3.8 [19.4] cm^2^) (*p* < 0.001 and 0.003, respectively). Also, within the limited resection group, the median size and area of tumors resected in the segmentectomy group was 5 [4.9] cm and 2.5 [4.2] cm^2^, respectively, whereas it was 1.5 [1.2] cm and 1.8 [3.2] cm^2^ in the wedge resection group (*p* = 0.01).

The in‐hospital mortality rate was 9 (3.6%) cases, consisting of seven primary cases of SCC (*n* = 5) and adenocarcinoma (*n* = 2), as well as two cases of metastatic breast cancer and soft tissue sarcoma. There was no statistically significant difference observed in terms of in‐hospital mortality when considering gender or type of lung cancer (*p* = 0.186 and 0.489 respectively). However, further analysis showed a significant association between both cigarette smoking and extensive resection and the in‐hospital mortality rate (*p* = 0.012 and 0.009, respectively).

Out of 232 patients, 15 individuals underwent two surgeries and one had three surgeries. In nine cases both lungs were involved at presentation and a second surgery on the opposite lung was done within a few months of the initial surgery. These cases included three metastatic soft tissue sarcomas, two osteosarcomas, one colon cancer, one PNET/Ewing family, one low and intermediate NET, one brain meningioma, and one large cell carcinoma.

There were four instances of tumor recurrence following limited resection, including cases of adenocarcinoma, soft tissue sarcoma, osteosarcoma, and germ cell tumor. Consequently, lobectomy was performed in the first three cases, and limited resection was conducted as a second surgery in the fourth case. Also, in two cases of PNET/Ewing family and osteosarcoma, the other lung became involved 2 and 3 years after the initial surgery, leading to a subsequent surgery on that side. The patient who underwent three surgeries initially had metastatic soft tissue sarcoma in the left lung, which was treated with limited resection. However, after 6 years, she presented with involvement of both lungs with the same tumor. Subsequently, two surgeries were performed, 4 months apart, involving limited resection on the right lung and lobectomy plus limited resection on the left lung.

## 4 Discussion

In this retrospective study, we conducted a comprehensive evaluation of the clinical, histopathological, and epidemiological features of lung cancer patients who underwent surgical resection in three referral hospitals in Southern Iran over a span of 12.5 years (2009–2022). Our results showed an overall increase in the rate of lung cancer surgeries during the study period. Males and patients aged 65 years or older were the predominant groups in both primary and secondary cases. Adenocarcinoma and soft tissue sarcoma were the most frequent pathologic subtypes in primary and secondary cases, respectively. Additionally, lobectomy emerged as the most commonly employed surgical technique for primary cases, while limited resection was utilized for metastatic lesions. In‐hospital mortality was significantly higher among smokers and patients who underwent extensive lung resection. To the best of our knowledge, this study represents the first report on lung cancer surgery statistics in Iran, illustrating the current status and shortcomings of lung cancer surgery in Southern Iran, thereby guiding policymakers and surgeons to take proper steps toward better management of this disease.

Our study focused on 232 lung cancer patients who underwent surgical resection during a 12.5‐year period at three main thoracic surgery centers in Shiraz, Southern Iran. In 2020, lung cancer was the second cause of cancer death in Iran, with 9071 recorded deaths [12]. Compared to our study, countries with screening programs have reported higher rates of operable cases [13]. In one Chinese study, conducted at a single university hospital, annual operation rates were 280, 376, 524 and 878 from 2016 to 2019 [13]. Another study conducted in a single institute in Korea reported a total of 2076 surgeries from 1990 to 2009 [14]. Similar study in Iceland reported 489 lung cancer patients undergoing lobectomy from 1991 to 2014 [15]. On the other hand, a study on lung cancer in the Middle East and North Africa region, like our study, found that the proportion of patients eligible for surgery was low, primarily due to the high rate of late‐stage diagnosis [16]. Although the number of our operated patients may appear small given the duration of the study, it reflects significant challenges in the region. The majority of lung cancer cases in the area are diagnosed at advanced stages, making surgery impractical. Timely surgical intervention was challenging, considering the lack of a lung cancer screening program, lengthy interval between diagnosis and treatment and high prevalence of regional opium addiction. Therefore, a comprehensive screening program, timely intervention and public health strategies are required to address these issues and improve lung cancer care in the area.

In our study, we observed a surge in annual surgical rates during the last year. This coincided with the coronavirus disease 2019 (COVID‐19) pandemic, and some patients were incidentally detected while undergoing assessment for COVID‐19 infection. The doubling number of patients eligible for lung cancer surgery, coupled with the absence of a significant decline in surgery rates in the preceding years, could be attributed to several factors. First, the increased use of diagnostic imaging for COVID‐19 led to the detection of asymptomatic lung cancers. Second, the rise in health‐seeking behaviors during the pandemic drove more patients with respiratory symptoms to seek medical attention, resulting in earlier diagnosis and intervention. Additionally, population aging has contributed to the increase in the overall number of lung cancer patients. This highlights the potential need for implementing screening modalities in our society. In recent decades, low‐dose computed tomography (LDCT) has emerged as a modality for lung cancer screening. Recent studies have indicated that LDCT may contribute to a higher detection rate among patients and, through early intervention, reduce both the mortality rate and burden of the disease [17]. Therefore, this screening program has been implemented among high‐risk populations in various regions such as North Amerika and Europe [18, 19]. In Iran, efforts are underway to evaluate the cost‐effectiveness of implementing this screening program and latest studies have shown promising results [20]. However, challenges such as limited resources persist in the region. In conclusion, considering the substantial mortality and disease burden associated with Lung cancer in Iran, it is essential for policymakers to evaluate the necessity of developing a lung cancer screening program.

Global cancer statistics in 2020 revealed a higher incidence rate of all cancers combined in men by 19% [1]. Consistently, men comprised the majority of our study population in both the primary and secondary groups. However, the gap between the two genders was narrow and did not show statistical significance. This issue could be attributed to the higher prevalence of secondhand smoking among Iranian females compared to males [21]. Moreover, women are prone to NSCLC due to a higher incidence of epidermal growth factor receptor mutations and estrogen effects [22]. Finally, higher doses and longer durations of smoking in Iranian men compared to women could result in more advanced stages of the disease [23]. Therefore, if not diagnosed early, it will increase the challenges for surgery and treatment.

The incidence of lung cancer in Iran over the past three decades was highest in the age group 70 years and older, followed by the age group of 50–69 [3]. Although the mean age of patients with primary lung cancer in our study was 56.70 ± 13.99, an increase in the proportion of patients above 65 years was observed in the second half of our study period, both in primary and secondary lung cancer patients. In a similar study conducted in Brazil, the mean age of patients was reported to be 63.96 ± 11.6, and during the study period, the number showed no significant rise [24]. Currently, palliative care is offered at higher rates to elderly patients with lung cancer, rather than possible curative surgery. This approach is mainly employed to avoid the risks associated with surgery, such as higher mortality and morbidity. However, recent literature suggests that with precise patient selection and integrative care, the elderly could benefit from surgery and achieve outcomes comparable to those of the younger patients [25]. Thus, the presence of a multidisciplinary team at centers with a thoracic surgery department is instrumental in making better operative decisions, providing better perioperative care, and achieving improved outcomes for elderly patients [26].

In the current study, the most common presenting symptoms among patients were found to be cough and dyspnea in both primary and secondary groups. In a similar study on surgical cases of lung cancer in Iceland, the most common presenting symptoms were cough (36.3%) and dyspnea (23.1%) [15]. However, a review by Latimer et al. highlighted that hemoptysis demonstrated the highest specificity for lung cancer [27, 28]. Xing et al. provided evidence indicating an association between the presenting symptoms of primary lung cancer with the histopathologic subtype and stage of the tumor. They found that patients with SCC were more likely to get symptomatic compared to patients with adenocarcinoma. Additionally, a symptomatic presentation was more likely in advanced stages [29]. In this regard, our study revealed that patients with low or intermediate NET significantly presented with hemoptysis. Nevertheless, still a significant number of patients remain asymptomatic even at stage IV of the disease, underscoring the importance of implementing screening programs for high‐risk populations [27].

Smoking is associated with an increased risk of postoperative morbidity and mortality [30]. Consistently, our study demonstrated a significantly higher in‐hospital mortality rate among smokers. Therefore, smoking cessation is considered an important preparation before pulmonary resection [31]. However, there is a controversy regarding the timing of cessation. Although Short‐term cessation immediately before surgery has not shown significant improvement in postoperative outcomes, the literature does not support delaying surgery for an extended period or denying it to current smokers solely to avoid postoperative complications [32, 33]. Given that even postoperative smoking cessation has shown favorable results in reducing the recurrence and metastasis rate, offering smoking cessation programs to patients at any time is beneficial [33].

In addition to smoking, the carcinogenic effect of opium consumption has become increasingly evident [34]. Opium consumption is correlated with an increased incidence of lung cancer, predominantly the SCC subtype [35]. Additionally, opioids can suppress respiratory symptoms and impede the timely detection of lung cancer cases [36]. Consequently, this delay often results in the deprivation of many patients from curative surgery. Moreover, in some patients detected at early stages, due to smoking or opium abuse, pulmonary function tests are impaired, once again depriving patients of surgery [37]. Given the high prevalence of opium consumption in Iran, one of the possible reasons for the low number of lung cancer patients eligible for surgery could be attributed to this issue.

NSCLC accounted for nearly 75% of our primary tumors, with adenocarcinoma being approximately twice as common as SCC. While SCC was previously the predominant histopathologic subtype, many studies in recent years have reported a higher incidence of adenocarcinoma in surgical cases. The proportion of surgical cases of adenocarcinoma from studies conducted in Japan (2010), Korea (2012–2016), Iceland (1994–2008), and Brazil (2011–2018) was reported to be 70.1%, 69%,57%, and 55.84% respectively [24, 38, 39, 40, 41]. An Indian study also reported adenocarcinoma as the most frequent subtype [42]. Results of a recent similar study from China indicated an increase in the proportion of adenocarcinoma cases from 55.5% in 2016 to 74.1% in 2019 [13]. There is currently a shift in the trend of lung cancer histopathologic subtypes worldwide, with an increase in adenocarcinoma and a decrease in SCC observed in many regions [40, 41, 43]. This transition is largely attributed to the changing smoking habits in these areas. However, the current histopathological trends in Iran remain unclear, and further nationwide studies with larger sample sizes are required to clarify this issue.

Surgery is the preferred treatment modality in clinical stages I and II of NSCLC and in patients with mentioned clinical stages who have pathological stage III [4]. In our study, the majority of our cases were at pathological stage I, followed by stages II and III, respectively. when comparing our findings with the reviewed literature, the frequency of our patients in each pathological stage was mostly similar to that of Korea, especially in the years 2007–2011, and Iceland between 1994 and 2008 [40, 43]. However, other studies have reported a higher rate of patients undergoing surgery with stage I disease [38, 40, 41, 44]. These findings suggest that we may be lagging behind universal trends, and it is necessary to take action towards improving patient detection in the future. Furthermore, besides the delayed diagnosis of patients, another possible factor contributing to patients being deprived of surgical treatment is the prolonged process from diagnosis to treatment in our region. During this time gap, the disease stage may change in some patients, rendering the tumor inoperable.

Lobectomy was the most frequently performed surgical procedure in our centers for the resection of primary lung tumors. A study on surgical cases of primary lung cancer in Korea from 1990 to 2009 revealed the following rates for surgical techniques: 26.9% for pneumonectomy, 11% for bilobectomy, 59.4% for lobectomy, and 2.6% for limited resections [14]. A recent study from Portugal reported 90.6% lobectomies for the period from 2012 to 2018 [38]. Another study from Japan reported that 22.7% of surgeries were limited resections [39]. In Iceland, the distribution of surgical techniques was 73.5% lobectomies, 14.9% pneumonectomies, and 11.6% limited resections [43]. Another study from a tertiary center in Brazil revealed that although the most performed procedure was lobectomy, the rate of segmentectomies increased during the study period [24]. While lobectomy is considered the gold standard method, recent studies have shown that segmentectomy can yield comparable oncologic results when performed on appropriately sized tumors with adequate free margins and sufficient lymph node dissection [45, 46]. On the other hand, pneumonectomy is associated with high rates of postoperative morbidity and mortality [47]. consequently, there is a global trend towards an increase in limited resections and a decrease in extensive resections [40, 47, 48]. However, among the surgical approaches for primary tumors in our centers, limited resections had the lowest rate, indicating that our surgeons may still adhere to the gold standard method or our patients may have been diagnosed at stages where segmentectomy is no longer considered safe. In conclusion, a comprehensive pre‐operative evaluation is necessary to adopt the most suitable surgical modality for each individual.

Surgical management is considered the preferred treatment for pulmonary metastasis, as complete resection of lesions offers a chance for potential cure [5]. Limited resection was the predominant surgical method adopted at our centers. Limited resection, particularly wedge resection, is the preferred surgical approach based on the parenchymal sparing principle and the high risk of disease recurrence [49]. However, a recent study by Prisciandaro et al. showed that in patients with pulmonary metastasis from colorectal cancer, anatomical resection was associated with improved recurrence‐free survival compared to non‐anatomical approaches [50].

The majority of lung cancer resections at our centers were performed using conventional open thoracotomy, whereas many centers worldwide have adopted minimally invasive methods like VATS from years ago [40, 41, 51, 52, 53]. Since its introduction, an increasing number of studies have confirmed that VATS is superior to open thoracotomy in many aspects. Patients operated with VATS method appear to have fewer post‐operative complications, shorter length of hospitalization, lower intraoperative bleeding, less chest tube drainage and better overall survival [7, 54, 55, 56]. however, the limited use of VATS in our centers could be attributed to inadequate infrastructures, high equipment costs, a shortage of trained surgeons, financial limitations and insurance coverage issues. Given that VATS is becoming the gold standard method [57], it is time for our centers to be equipped with VATS instruments, for our surgeons to master VATS surgery and for health insurances to start covering VATS for lung cancer surgeries.

Similar to our results, colorectal cancer, soft tissue sarcoma, osteosarcoma, renal cell carcinoma, and head and neck tumors are reported as the most common cancers that tend to metastasize to the lung [58]. Pulmonary metastasectomy is now widely practiced as part of the multidisciplinary management of metastatic disease in selected patients. In cases of metastatic colorectal cancer, operative resection carries a favorable prognosis among patients with isolated unilateral pulmonary metastasis, no lymph node involvement, and normal carcinoembryonic antigen levels [59]. Similarly, surgery has shown to be the best treatment option for lung metastasis originating from soft tissue and osteogenic sarcomas by far [60, 61]. Nevertheless, results vary among tumors of different origins, and further randomized clinical trials are needed to confirm the benefits of this practice for each pathology.

Although surgery remains a cornerstone in early‐stage lung cancer treatment, adjuvant or neoadjuvant therapy may be necessary in some cases to improve outcomes [62]. At our centers, patients with locally advanced carcinoma and high‐grade sarcoma received neoadjuvant chemotherapy prior to surgery. Evidence suggests that neoadjuvant therapy can eradicate micro metastasis, shrink tumors, enhance tumor resection, and improve overall outcomes [63]. Additionally, recent research has demonstrated promising results with the integration of targeted therapy and immunotherapy [41, 62]. Specifically, immunotherapy has shown significant improvements in survival rates compared to chemotherapy alone [64]. Therefore, a multidisciplinary approach is necessary to adopt the best treatment strategy and optimize outcomes in lung cancer patients.

For a long time, the burden of lung cancer was predominantly borne by developed nations [65]. However, due to epidemiological changes, developing countries like Iran are expected to face an increase in the incidence of this disease [3]. To effectively manage the evolving situation, policymakers and healthcare professionals should be prepared in advance [3]. One of the most important issues that should be a top priority for policymakers is addressing health disparities. These disparities at both national and subnational levels significantly influence cancer care [66]. Research indicates that higher‐income countries and individuals with better socio‐economic status have more favorable outcomes [67]. Compared to other developed regions, access to advanced surgical technologies and some medications is limited in Iran. Moreover, the lack of an established screening program and a structured referral system leads to late diagnosis. Similarly, research suggests that countries in the Middle East and North Africa region face similar challenges in prevention, diagnosis, and treatment of lung cancer [16]. Within Iran, people in rural areas have less access to specialized care. Additionally, financial barriers and poverty play a significant role in both health‐seeking behaviors and accessing care. Policymakers should effectively plan to reduce these disparities by improving social, cultural, geographical, economic, and informational access to healthcare, ensuring that all individuals receive equitable care. Another critical issue is the implementation of preventive measures. Since tobacco products are the primary cause of lung cancer, effective policies aimed at reducing their use can significantly control the disease [68]. While many Western countries have successfully controlled smoking prevalence, resulting in a decline in lung cancer incidence, developing countries like Iran, as well as those in the Middle East and North Africa region, have not achieved similar progress [16]. We believe that investing in healthcare infrastructure, implementing nationwide screening programs, enhancing drug availability, improving insurance coverage for cancer care, increasing health literacy, implementing stronger smoking cessation policies, and developing a more comprehensive referral system would significantly improve lung cancer care in Iran.

Our study was subject to some limitations primarily due to its retrospective nature. Consequently, some data pertaining to the patient's presentation at diagnosis, smoking dosage (pack‐year), pathological staging, and imaging reports were missing. Additionally, we were unable to obtain access to the patient's pulmonary function tests and detailed information about additional therapies apart from surgery. Moreover, patients were not followed to determine the 30‐day mortality rates and 5‐year survival rates. Finally, since the present study exclusively focuses on patients who underwent surgical treatment, it cannot be regarded as representative of the entire population of lung cancer patients in our region.

In light of the identified limitations, the following recommendations are proposed to improve the quality and scope of future studies. Adopting a prospective study design would ensure more detailed and accurate data collection. Additionally, implementing a comprehensive regional lung cancer registry and electronic health records is essential for better tracking of lung cancer trends and enhancing data accuracy and accessibility. Long‐term follow‐up studies are crucial for assessing long‐term survival rates. Finally, conducting comprehensive research on non‐surgical patients and performing similar studies in other regions of Iran would provide a broader understanding of lung cancer management and facilitate regional and national comparisons.

## 5 Conclusion

It appears that the number of lung cancer surgeries is increasing in Southern Iran, particularly among men, who represent the most common cases requiring surgical intervention for lung malignancies. The majority of surgical interventions were performed on patients with lung adenocarcinoma, metastatic soft tissue sarcoma, and metastatic colon cancer. At our centers, the preferred surgical techniques for primary and secondary lesions were the gold standard methods of lobectomy and limited resection, respectively. It is expected that by reducing smoking habits and minimizing the use of extensive resections, there will be an improvement in the short‐term postoperative survival of patients.

## Author Contributions

A.A. and R.S. designed the study. S.D. collected the data and drafted the manuscript. R.S. and S.D. analyzed the data. A.R. and M.J.F. assisted with the pathological diagnosis features, while B.Z., P.M., and A.A. provided valuable insight the surgical features. A.A. supervised the project. A.R., M.S.R., and R.S. revised the manuscript. All authors read and approved the final manuscript.

## Ethics Statement

The present study was approved by the Ethics Committee of “Shiraz University of Medical Sciences” and all experiments were performed in accordance with relevant guidelines and regulations. Based on the retrospective nature of the study, an informed consent waiver was approved by the Ethics committee of Shiraz University of Medical Sciences. Patients' information was anonymized before analysis and confidentiality was assured by the researcher.

## Consent

The authors have nothing to report.

## Conflicts of Interest

The authors declare no conflicts of interest.

## Data Availability

All data regarding this study has been reported in the manuscript. Please contact the corresponding author if you are interested in any further information.
